# Understanding and leveraging placebo and nocebo effects in perioperative care: a cross-sectional survey of German-speaking anesthesiologists

**DOI:** 10.1186/s12871-025-03579-w

**Published:** 2026-01-05

**Authors:** Johannes Wessels, Robert Jan Pawlik, Claudia Foerster, Joachim Erlenwein, Sven Benson, Wiebke Sondermann, Sigrid Elsenbruch, Jana Aulenkamp

**Affiliations:** 1 Department of Anesthesiology, University Medical Center Hamburg Eppendorf, Hamburg, Germany; 2 Department of Neurology, Center for Translational Neuro- and Behavioral Sciences (C-TNBS), University Hospital Essen, University Duisburg-Essen, Essen, Germany; 3 German Pain Center Mainz, Mainz, Germany; 4 Department of Anesthesiology, University Medical Center Goettingen, Goettingen, Germany; 5 Scientific Group and Section on Pain Medicine, German Society of Anesthesiology and Intensive Care Medicine, Nuremberg, Germany; 6 Institute for Medical Education, Center for Translational Neuro- and Behavioral Sciences (C-TNBS), University Hospital Essen, University of Duisburg-Essen, Essen, Germany; 7 Department of Dermatology, Venereology and Allergology, University Hospital Essen, University Duisburg-Essen, Essen, Germany; 8 Department of Medical Psychology and Medical Sociology, Ruhr University Bochum, Bochum, Germany; 9 Department of Anesthesiology and Intensive Care Medicine, University Hospital Essen, University Duisburg-Essen, Essen, Germany

**Keywords:** Expectation, Open-label placebo, Suggestion, Non-pharmacological intervention, Communication

## Abstract

**Background:**

Placebo and nocebo effects, beneficial or adverse responses shaped by patient expectations, modulate perioperative outcomes including pain, opioid requirement, recovery, and complication risk. Although harnessing these mechanisms may improve patient care, their routine use by anesthesiologists remain unexplored. This study systematically assessed anesthesiologists’ knowledge, perceived relevance, and clinical use of placebo and nocebo effects, to identify educational needs and opportunities for enhanced patient care.

**Methods:**

An observational cross-sectional online survey, was conducted in April-May 2024 among German-speaking anesthesiologists, covering diverse clinical settings and career stages. The questionnaire evaluated theoretical knowledge, perceived relevance, clinical application, attitudes toward placebo interventions and feasibility of specific strategies to enhance placebo responses and minimize nocebo effects in clinical practice.

**Results:**

Of 650 respondents (436 complete), self-reported knowledge of placebo effects was moderate (median = 6; 0–10), and lower for nocebo effects (median = 5; *p* < 0.001). Placebo mechanisms were deemed most clinically relevant in pain management (78%) and palliative care (82%). While 72% considered the use of placebo effects as acceptable and 18% essential, only 35% reported deliberate application of placebo knowledge, and 23% used nocebo mitigation strategies. Contrastingly, 92% routinely employed communication strategies to shape expectations. Placebo utilization included open-label (27%), impure (e.g., subtherapeutic analgesics; 58%), and deceptive placebos (48%).

**Conclusion:**

Anesthesiologists frequently integrate expectation-based interventions widely, however gaps in theoretical knowledge and deliberate systematic application remain, particularly regarding nocebo effects. Targeted education and evidence-based guidelines may foster ethical, systematic integration of placebo and nocebo effects in perioperative care and improve patient care and safety in anesthesiology.

**Supplementary Information:**

The online version contains supplementary material available at 10.1186/s12871-025-03579-w.

## Introduction

 Medical treatment outcomes rely not only on pharmacological or technical interventions, but also on individual psychological mechanisms [1]. Placebo studies have demonstrated that patients’ expectations and their prior experiences can exert a substantial influence on treatment outcomes, either positively (placebo) or negatively (nocebo) [2–5]. For instance, the analgesic effect of remifentanil can be enhanced or diminished by clinician communication [3]. Anesthesiologists, who guide patients through vulnerable perioperative phases, are uniquely positioned to influence the psychological state of a patient [6, 7]. However, it remains unclear how well they understand, prioritize or intentionally apply placebo and nocebo effects in clinical routine.

Placebo effects, defined as improvement in symptoms or clinical outcomes occurring independently of a treatment’s active pharmacological components, are mediated by neurobiological pathways involving endogenous opioids, dopaminergic, and cannabinoid systems [8–12]. Meta-analyses support placebo effects across various physiological systems, including respiratory, cardiovascular, immune, and pain processing [3, 13, 14]. Harnessing positive expectations has demonstrated efficacy in reducing pain by up to 46%, decreasing opioid consumption by 34%, and accelerating recovery [15–18], directly mitigating risks and hospital-acquired complications [19]. 

Conversely, nocebo effects, driven by negative expectation and prior experience, can harm patients by exacerbating symptoms, increasing pain sensitivity, triggering adverse events, delaying recovery, or reducing treatment efficacy or adherence [3–23]. Elements of routine clinical communication, such as procedural warnings (e.g., “This will hurt”) or negative suggestions, can elevate postoperative nausea rates and analgesic demand, highlighting preventable risks to patients [20, 21]. 

Given that expectation effects are inherent to most medical treatments, it is unsurprising that clinicians seek to harness their therapeutic potential. This encompasses the use of placebos (e.g., inert substances) or impure placebos (e.g., sub-therapeutic doses of active drugs), which appear more common than often assumed [24–26]. However, such practices raise ethical concerns, particularly regarding patient autonomy and informed consent.

Despite growing interest in incorporating placebo and nocebo mechanisms to enhance safety, minimize harm, and leverage non-pharmacological strategies, [2, 5, 7] evidence regarding anesthesiologists’ knowledge and application remains scarce. To address this gap, we conducted a cross-sectional online survey to assess anesthesiologists’ understanding, perceived relevance and clinical use of placebo and nocebo effects, including their attitudes toward placebo interventions. Thereby, we intended to promote dialogue on the ethical and practical integration of these mechanisms in routine clinical practice and to guide development of educational frameworks aiming to contribute to enhanced patient care and safety in anesthesiology.

## Methods

This cross-sectional survey was conducted in collaboration with the Scientific Group and Section on Pain Medicine of the German Society for Anesthesiology and Intensive Care Medicine (Nuremberg, Germany) with ethical approval (Reference No. 25-12338-BO). This manuscript followed current recommendations for reporting on survey studies [27]. 

The questionnaire was devised by a multidisciplinary team comprising experts in anesthesiology, psychology, medical education, and pain medicine, building upon previous surveys on expectancy effects in dermatology and pediatrics [24, 25]. General questions regarding expectancy effects were adopted from this prior surveys, while discipline-specific questions were adapted to anesthesiology through an interdisciplinary consensus to ensure content alignment and maintain construct and content validity. After pilot testing and feedback from colleagues, the survey consisted of 36 questions (for complete questionnaire see Supp. 1), distributed across nine sections, using quantitative (e.g., multiple-choice and numeric rating scales) and qualitative methods to examine placebo and nocebo effects, alongside sociodemographic data. This study represents the initial, quantitative phase of a larger mixed-methods project; subsequent qualitative phases (e.g., content analysis) will build on these findings to deepen understanding and guide implementation in clinical practice.

Participants, all anesthesiologists were invited to self-assess their knowledge of placebo and nocebo effects using a numerical rating scale (NRS; 0 = no knowledge at all, 10 = best possible knowledge), indicate knowledge sources, and express interest in further information. Participants rated their agreement with specific statements designed to assess their understanding of these effects on a scale of agreement (NRS; 0 = completely false, 10 = completely true), adapted from Krefting et al. [26]. Participants were asked to report the relevance of placebo effects in clinical areas and to evaluate the feasibility of implementing communication strategies designed to maximize placebo and minimize nocebo effects in routine practice. Clinical application and symptom-specific uses were assessed, inspired by Faria et al. [25]. Participants disclosed their utilisation of deceptive, impure (treatments intended solely to elicit a placebo response, such as underdosing or incorrect indications) or open-label placebos (OLP; patients are aware of the treatment’s inert nature), including substances administered.

The survey was conducted anonymously using the online survey tool LimeSurvey (LimeSurvey GmbH, Hamburg, Germany; http://www.limesurvey.org) and data were assessed in April and May 2024. All participants provided written informed consent following written information about the study aims (see Supplement 1); participation was voluntary and could be terminated at any time. To minimize multiple participation in this web-based survey, session-based participation restrictions were implemented using cookies. The survey link was sent out by the society DGAI two times to all *N* = 12,713 members via email, and 39.7% (5,059/12,713) of the recipients opened the email. In total, 650 responses were received, of which 436 provided complete answers and were included in the analysis. Given the total number of 12,713 anesthesiologists who received the survey invitation, this represents an estimated response rate of 5.1%, comparable with other surveys in this field [24, 25]. 

Descriptive statistics were calculated as percentages for categorical data and medians with interquartile ranges (IQR) for non-normally distributed data. Unless specified otherwise, analyses refer to *N* = 436. Group differences were analyzed using chi-square tests for categorical variables and Kruskal-Wallis tests for metric data, followed by post-hoc Dunn tests where applicable. For analysis, senior physicians and department heads were combined, as were respondents in ambulatory care, retired professionals, and other roles. Spearman’s correlation was examined for association between reported knowledge and perceived relevance. Statistical analyses were conducted using SPSS Statistics (version 29), and figures were generated using GraphPad Prism (version 10). No statistical weighting was applied.

## Results

Of the 436 responses, 421 participants indicated their gender, with the majority identifying as male (55.1%, Table 1). Regarding professional status, most held senior positions (39.8%, e.g., senior physician or head of department) and the primary clinical focus specialty of the anesthesiologists reported was anesthesia (Table 1).

**Table 1:** Overview of participants‘ characteristics

	Total
*N*	436
Gender Female Male Diverse	421 188 (44.7%) 232 (55.1%) 1 (0.2%)
Age, years, mean ± SD; [range]	46.4 ± 12.0; [25–80]
Professional Position Trainee Specialist Senior Consultant Department Head Ambulatory care Retired Other	432 94 (21.4%) 138 (31.4%) 123 (28.0%) 51 (11.6%) 17 (3.9%) 6 (1.4%) 7 (1.6%)
Clinical Focus Anesthesia Intensive Care Medicine Emergency Medicine Pain Medicine Palliative Medicine Other	432 304 (69.1%) 58 (13.2%) 11 (2.5%) 39 (8.9%) 9 (2.0%) 15 (3.4%)

Unless otherwise stated, values are given in numerical and percentage format

### Understanding of placebo and nocebo Effects

**Fig. 1:**
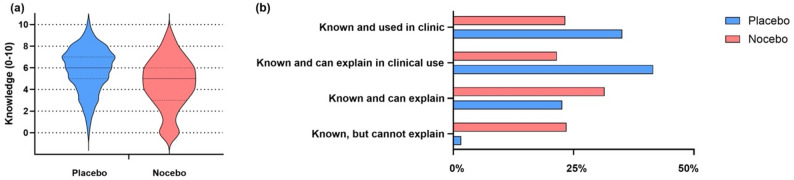
Self-assessed knowledge of placebo and nocebo effects for all participants (**a**) and reported confidence of respondents about their knowledge on placebo (blue bars, *N* = 433) and nocebo effects (red bars, *N* = 403) in percentages (**b**), a) placebo: median 6, IQR (5 to 7) and nocebo: 5 IQR (3 to 7). Statements had to be rated on a NRS 0–10 (0 = no knowledge at all, 10 = best possible knowledge), Violin plots illustrate the answer density, median and first and third quartiles

Almost all participants (99.3%; 433/436; Fig. 1a) indicated having some knowledge of placebo effects with a median of 6 [IQR 5 to 7]. Knowledge levels varied significantly by professional role (H(3) = 19.516, *p* < 0.001), with trainees reporting significantly lower knowledge than senior physician doctors (*p* = 0.001). A gender difference was also observed (H(2) = 6.240, *p* = 0.044), with female participants rating their knowledge lower than male participants (5.53 vs. 6.06; *p* = 0.038)

For nocebo effects, 92.4% (403/436) reported some knowledge, with a median of 5 [IQR 4 to 6]. Participants rated their knowledge of nocebo effects lower as for placebo effects (*p* < 0.001). Similar to placebo, nocebo knowledge differed by professional role (H(3) = 21.900, *p* < 0.001), with trainees reporting lower levels compared to physicians (*p* = 0.014) and senior physicians (*p* = 0.001) but no differences by gender was found (H(2) = 5.548; *p* = 0.062).

**Fig. 2:**
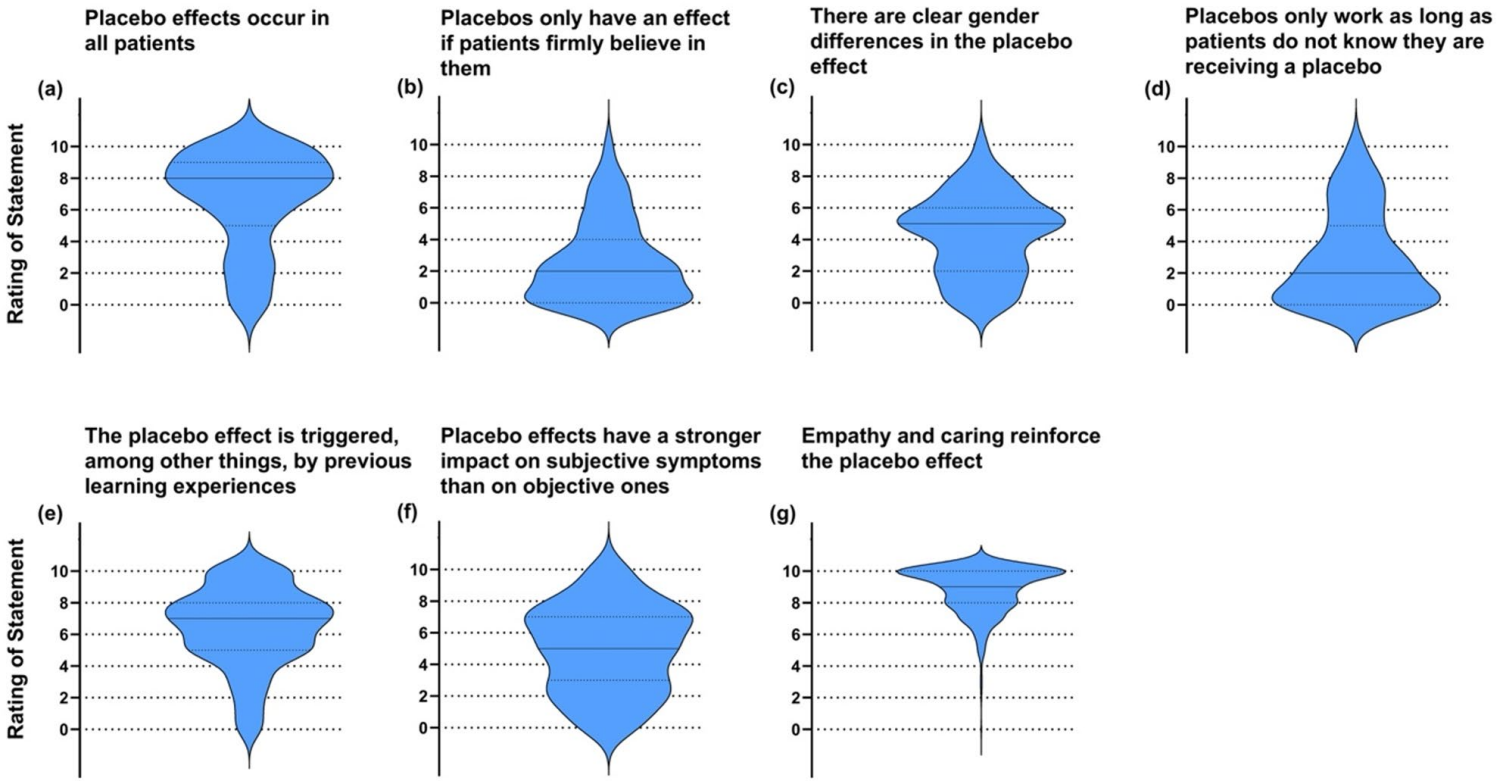
Responses of participants on the closed questions regarding placebo effects. Panels visualize statements that had to be rated on a NRS 0–10 (0 = completely false to 10 = completely true). Violin plots illustrate the answer density, median and first and third quartiles

**Fig. 3:**
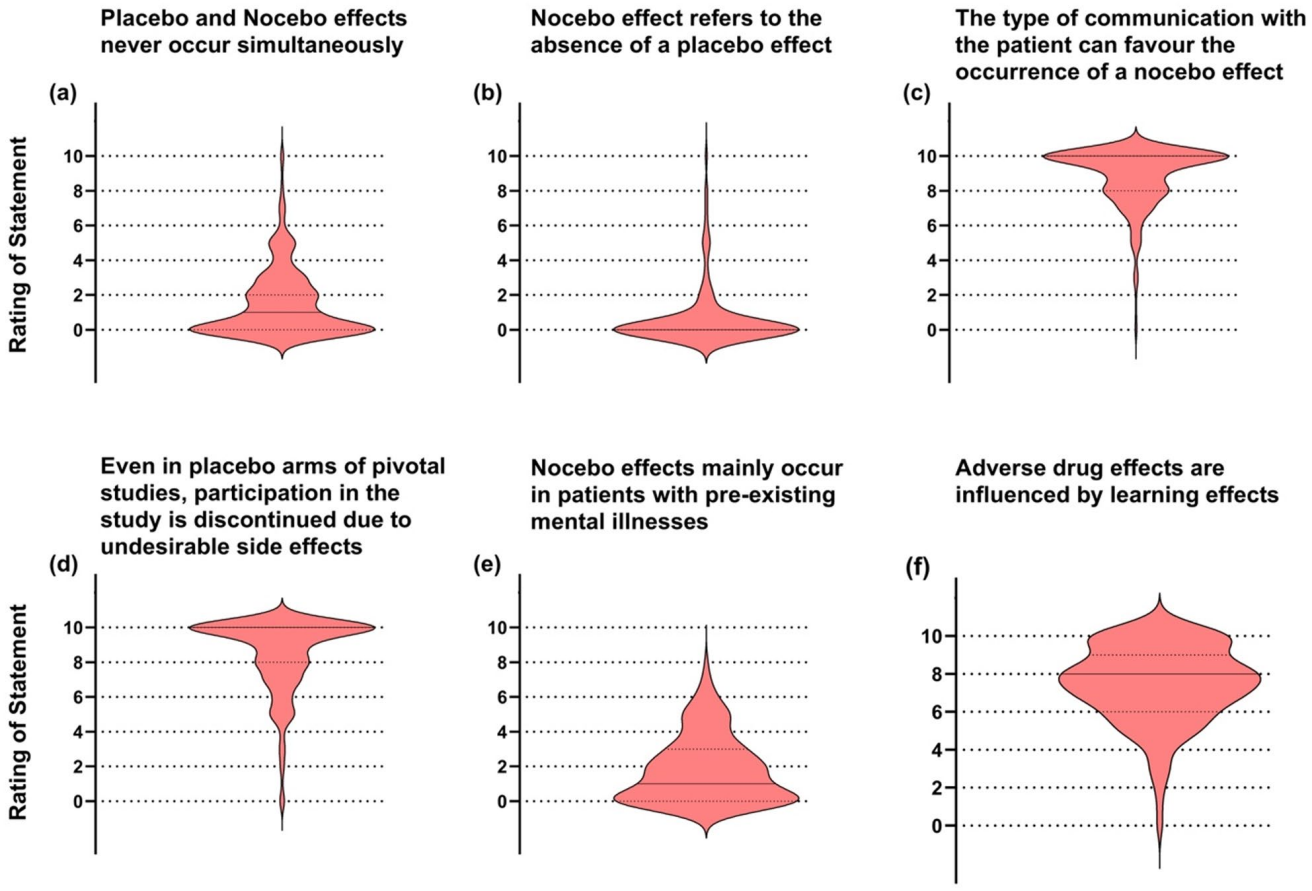
Responses of participants on the closed questions regarding nocebo effects. Panels visualize statements that had to be rated on a NRS 0–10 (0 = completely false to 10 = completely true). Violin plots illustrate the answer density, median and first and third quartiles

Participants’ knowledge of placebo effects was assessed using closed-ended questions, resulting in a wide distribution of responses (Fig. 2). Highest agreement was found for (a) placebo effects occur in all patients, (e) learning experiences as a trigger, and (g) reinforcement through empathy and care. Uncertain or mixed ratings were given for (c) gender differences and (f) stronger impact on subjective vs. objective symptoms. Statements (b) requiring belief in effect and (d) effectiveness only when undisclosed received consistently low agreement. Similarly, knowledge of nocebo effects was evaluated (Fig. 3). Strong agreement was observed for (c) communication type as a factor, (d) adverse effects in placebo arms, and (f) influence of learning on adverse effects. In contrast, participants disagreed with (a) mutual exclusivity of placebo and nocebo, (b) nocebo as mere absence of placebo, and (e) restriction to patients with mental illness.

### Confidence of knowledge of placebo and nocebo effects

Nearly all participants who indicated some knowledge considered it sufficient to explain placebo effects to others (98.4%; 426/433; Fig. 1b). However, only 34.9% of the participants agreed that they actively apply their knowledge of placebo effects in clinical practice, exhibiting notable differences by professional role (χ²=15.809, *p* = 0.001). Notably, only 21.5% of trainees reported active application, compared to 44.5% of senior consultants or department heads. Among the 403 participants familiar with nocebo effects, 23.5% agreed with the statement, “I have heard of the nocebo effect but am unable to explain it” (χ²=8.777, df = 3, *p* = 0.032; Fig. 1b), with trainees showing the highest agreement (35%).For further information regarding the sources of knowledge on expectation effects and the use as well as the feasibility of communication to enhance placebo effects, please see supplemental material 2.

### Use and relevance of placebo effects in clinical practice

Most respondents considered the use of placebo effects generally acceptable (71,6%), with 18.1% asserting its use to be essential and only 9.9% considering it acceptable in rare cases, and 0.5% never. Clinical improvements attributable to placebo effects were occasionally observed by 50.2% of respondents and frequently by 31.9%, while only 1.8% reported never observing such effects.

Placebo effects were rated as most relevant in pain medicine (9 (IQR 8 to 10) and palliative care 8 (IQR 7 to 10, anesthesia: 6 (IQR 3 to 8); intensive care: 6 (IQR 3.25 to 8): emergency medicine: 6 (IQR 3 to 8). Relevance in emergency medicine varied significantly by professional role (H(3) = 14.421, *p* = 0.002), with lower ratings from retired and ambulatory physicians compared to trainees (7 vs. 2.5; *p* = 0.002) and senior physicians (7 vs. 2.5; *p* = 0.002). Higher self-rated knowledge of placebo and nocebo effects was positively correlated with the perceived relevance of placebo effects in anesthesia (placebo: rho = 0.306; nocebo: rho = 0.360; both *p* < 0.001).

Placebo effects were considered most pertinent to specific tasks and contexts, including the management of chronic pain, recovery room care, and informed consent for anesthesia (Table 2). Symptom-specific relevance was highest for anxiety, followed by chronic and acute pain (Table 2).

**Table 2:** Relevance of placebo effects for various clinical activities and symptoms, as indicated by participants, listed in descending order. The percentages indicate the proportion of participants who consider each activity or symptom to be relevant for placebo interventions. Multiple answers up to five were possible

Clinical Activity	Reported Relevance	Symptom	Reported Relevance
Treatment of Chronic Pain	77.5%	Anxiety	89.4%
Anesthesia Recovery Room	57.8%	Chronic Pain	82.1%
Anesthesia Consultation	49.1%	Sleep Disorders	50.9%
Treatment of Acute Pain	45.2%	Acute Pain	51.4%
Anesthesia Induction	42.4%	Nausea	44.0%
Postoperative Visit	32.6%	Depression	27.3%
Regional Anesthesia	30.0%	Gastrointestinal symptoms	25.7%
Interventions/Punctures	28.2%	Fatigue	19.5%
Stationary Ward Rounds	25.5%	Addiction	8.3%
Emergency Care	25.2%	Withdrawal Symptoms	7.1%
Family Consultations	24.1%	Delir	3.0%
End of anesthesia	3.0%	Infection	0.7%
Emergency room treatment	1.1%		

### Use of placebo interventions in clinical practice

**Fig. 4:**
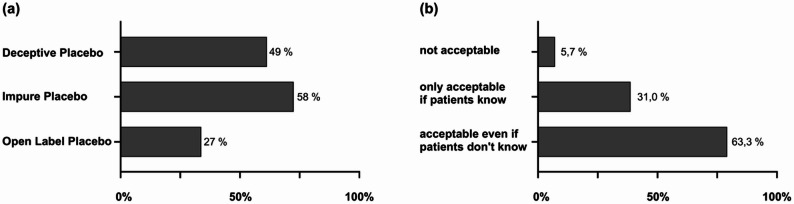
Usage of placebo interventions (**a**) and participants’ opinion regarding the use of placebos in clinical practice (**b**). The figures given in the diagrams are percentages

Placebo use was widely accepted by participants. Notably, 49.3% (210/426, Fig. 4a) reported covertly administering a placebo at least once (e.g., saline infusion labelled as a painkiller) with no gender difference (χ²=2.541, df = 2, *p* = 0.281), and 75.7% (317/419) had observed colleagues engaging in similar practices. However, only 7.6% (16/210) of those administering placebos reported informing patients in advance about possible use.

Furthermore, 57.6% (245/425) of participants reported prescribing or administering impure placebos, defined as treatments intended solely to elicit a placebo response, such as underdosing or incorrect indications. The most utilized substances were saline solutions (48.6%, 119/245) and analgesics (34.7%, 85/245), with no instances of antibiotic use reported for this purpose. A total of 26.6% (113/425) of respondents reported prescribing or administering open-label placebo medications. Usage also varied significantly by professional role (χ²=18.591, df = 3, *p* < 0.001), with senior consultants reporting the highest usage (35.3%).

Most participants considered both covert and open administration of inert substances ethically acceptable; 31% advocating for use only with patient knowledge, and 5.7% deeming them entirely unacceptable (Fig. 4b). Also, most respondents (86.8%) indicated a willingness to utilize open-label placebos with greater frequency, contingent upon evidence of clinical benefit in anesthesiology care and confirmation by professional guidelines. Notably, 83% expressed interest in learning more about placebo and nocebo effects.

## Discussion

This study provides the first evaluation of anesthesiologists’ knowledge, perceived relevance, and clinical use of placebo and nocebo effects. Although these mechanisms have been investigated in other medical disciplines [5, 24, 25, 28], anesthesiology has received comparatively less attention. Our findings reveal a paradox: while most participants routinely employed expectation-informed communication, only one-third explicitly reported conscious placebo application and even less used nocebo-reducing strategies. This highlights a knowledge-practice gap that may restrict implementation of evidence-based expectation management. However, most respondents expressed willingness to deepen their understanding.

### Knowledge of placebo and nocebo effects

Most respondents reported moderate familiarity with placebo and nocebo effects, with greater self-reported knowledge of placebo mechanisms. This mirrors literature that historically emphasizes placebo over nocebo research. Knowledge sources varied by career stage, with trainees more often learned about expectation effects during medical school, compared to dermatology (see supplemental material 2 [25]). This may reflect the focus on subjective outcomes such as pain and anxiety in anesthesiology and evolving educational priorities [25]. Interestingly, despite lower self-assessed knowledge, participants’ responses regarding mechanism of placebo and nocebo effects were correct and aligned with state-of-the-art scientific consensus, suggesting either an underestimation of expertise or limited conscious application. Minor misconceptions remained, particularly regarding the greater impact of placebo effects on subjective versus objective symptoms, a concept supported by substantial evidence (for more detailed discussion see Krefting et al. 2023 [26] ). Despite routine expectation-informed communication and their highly rated feasibility (see supplemental material 2), conscious placebo application and nocebo mitigation remained limited, reflecting intuitive rather than intentional practice that may restrict systematic implementation [28]. Given robust evidence that expectation management reduces pain, anxiety and even opioid consumption, [17, 21–23] explicit expertise is crucial [29]. Notably, reported knowledge scores positively correlated with reported clinical application, supporting the notion that knowledge affects subsequent clinical action.

These results highlights a compelling need for targeted educational initiatives enhancing implementation and reflective practice. Training interventions reduce patient anxiety and even procedural time, [21, 22] while e-learning and simulation programs, including those conducted within our research group, have improved clinicians’ confidence and skills [30, 31] and may further boost anesthesiologists’ confidence and self-reflection capabilities. Encouragingly, most participants expressed willingness to deepen their understanding, suggesting educational initiatives would be well-received and impactful.

### Relevance of placebo-effects in clinical practice

Participants deemed placebo effects acceptable and reported improvements attributable to placebo effects, reflecting both familiarity with and ability to detect these mechanisms in daily practice [1, 3, 32]. Placebo effects were rated as the most relevant in pain management and palliative care, reflecting established multimodal approaches and based on neuroimaging evidence of µ-opioid activation comparable to analgesics [10, 16]. Despite compelling evidence supporting placebo effects across diverse areas, from perioperative care to emergency medicine, [1, 16] placebo effects remain under-recognized in certain perioperative phases such as informed consent and anesthesia induction, where communication significantly influences outcomes and patient safety [6, 21]. The emphasis on chronic over acute pain applications, despite robust evidence in both contexts, likely reflects routine non-pharmacological strategies in chronic pain management. Educational initiatives should emphasize expectation management across all perioperative phases, particularly during premedication conversations and anesthesia induction [1, 20, 33]. 

### Placebos in clinical routine

Nearly half of participants admitted using deceptive and impure placebos, such as underdosing, with only 7.5% disclosing such use to patients. This paternalistic approach, in which physicians [24, 25] assume authority over patients’ needs and decisions, risks undermining patient autonomy, and perpetuate stigma among vulnerable populations, [34] even if intended to maximize therapeutic outcomes. The widespread acceptance of deceptive practices (deemed ethically acceptable by many respondents) [25, 34] contrasts with contemporary consent standards emphasizing transparency and shared decision-making.

More encouraging was the 27% prevalence of open-label placebo (OLP) use—higher than reported in other specialties [24] —particularly among experienced anesthesiologists, which may be due to greater confidence in communication, and increased comfort when discussing sensitive or ethically complex topics. This suggests growing recognition of ethical alternatives preserving therapeutic benefit while respecting patient autonomy. Evidence supporting OLP efficacy demonstrates similar analgesic benefits to deceptive placebos while adhering to informed consent principles [16]. The high disagreement with statements that placebo effects require patient deception indicates sophisticated understanding of transparent application potential, although ambiguities in the definition of “inert substances” within the survey could have influenced responses.

Reported prescriptions predominantly involve saline solutions or non-essential medications like vitamins, rather than inert substances such as sugar pills [24]. Ethical considerations, such as the categorization of saline—commonly used in volume therapy—as a placebo, remain contentious due to its dual roles as both inert and functional. Further patient-centered research (e.g. Wessels et al. [35]) and guidelines are needed to support their broader clinical adoption.

Gender differences in knowledge and use of expectation effects were small. Male participants reported higher self-rated knowledge of placebo effects, echoing findings from other fields and suggesting possible underestimation among female clinicians [25]. However, no gender differences were observed in the reported clinical application of placebo interventions.

### Limitations and perspective

Given the modest response rate, study limitations include a potential selection bias toward clinicians with a preexisting interest in placebo and nocebo mechanisms, which may limit the generalizability of our findings. Senior physicians were overrepresented, while trainees were likely underrepresented, possibly skewing the results toward a more experienced perspective. Even among this experienced group, knowledge and application remained limited, suggesting broader gaps across the anesthesiology community. To minimize survey burden, we did not ask about participants’ institutions; however, distribution via the DGAI ensured broad representation across professional groups. Furthermore, some survey items (e.g. regarding open prescription of placebos), may have led to misinterpretation, possibly affecting the reliability. The reliance on self-reported data carries risk of social desirability and recall bias, potentially inflating knowledge and application rates and exaggerating the knowledge–practice correlation. Future studies should triangulate self-reports with objective measures such as validated knowledge tests, clinical vignettes, or observational audits of communication. While the present study concentrated on German-speaking anesthesiologists, the results appear to be consistent with international surveys in various healthcare systems, which report similar patterns of placebo use (45–86%) [5, 26, 28] moderate knowledge, and limited awareness of nocebo mechanisms. International experts concur that training healthcare professionals in expectation management is a universal priority across cultural contexts [5]. These similarities suggest that, despite possible differences in training models, communication norms, and patient expectations, the findings are likely to be relevant to the broader international anesthesiology community. However, clinical application of expectation effects may vary across perioperative subspecialties (e.g., pediatric, cardiac, regional anesthesia), and generalization should be made cautiously. Finally, the large and demographically diverse sample provides a unique, novel and robust dataset, offering first insights into knowledge and clinical use of placebo and nocebo in anesthesiology [36].

## Conclusion

This survey highlights that anesthesiologists routinely apply communication strategies to shape expectations, yet their self-reported understanding of placebo mechanisms remains moderate and nocebo awareness is notably limited, revealing a significant knowledge-practice gap. Expectation effects were considered highly relevant in clinical practice, particularly in chronic pain and palliative care, where positive communication is integral to treatment. However, the widespread use of deceptive placebos combined with low disclosure rates raises ethical concerns and argues for greater emphasis on transparent, autonomy-preserving approaches [37].

Together, these findings underscore the need for structured education in expectation management, including communication skills, nocebo mitigation, and patient-centered consent for clinical improvement. Deeper integration of placebo and nocebo mechanisms into clinical routine, supported by guidelines that embed expectation-based interventions throughout perioperative care, could help bridge this gap, enhance patient safety, and inform non-pharmacological strategies beyond anesthesiology.

## Supplementary Information

Supplementary Material 1.

Supplementary Material 2.

## Data Availability

The data will be made openly and publicly available on OSF after publication of our article.
