# Supplementary material for: Understanding and leveraging placebo and nocebo effects in perioperative care: a cross-sectional survey of German-speaking anesthesiologists
Source: BMC Anesthesiol. 2026 Jan 5;26:70. doi: 10.1186/s12871-025-03579-w (PMC12853822; doi:10.1186/s12871-025-03579-w)
Supplement: Supplementary file 2 — Supplementary Material 2. [file 12871_2025_3579_MOESM2_ESM.docx]

**Supplemental Material**

**Additional results:**

*Sources of knowledge about expectation effects*

Participants reported various sources of knowledge about placebo and nocebo effects. For placebo effects, the most frequently cited sources were medical school (61.9%), scientific literature (58.2%), discussions with colleagues (52.7%), continuing education programmes (45.5%), and specialist training during residency (43.6%). Media was a less common source (21.0%). Trainees predominantly credited medical school (88.2%), whereas specialists and senior physicians more often cited literature (67.6%) and continuing education (56.1%). For nocebo effects, the primary sources were literature (54.1%), training programmes (46.7%), and discussions with colleagues (41.2%). Medical school was less commonly reported (35.2%), with trainees making up most of this group (67.5%).

*Use of communication to enhance placebo effects*

Participants indicated frequently using communication methods to enhance placebo effects and mitigate nocebo effects (median 8, IQR 6 to 9). The most frequently employed communication methods were positive framing (64%), expectation management (62.8%), and patient education (57.3%; Fig. 4). Only 11.5% of respondents indicated proactively disclosing information to mitigate nocebo effects. Communication methods were rated as feasible by 91.5% of participants. Among those who found them unfeasible (8.5%, 36/436), lack of confidence (55.6%, 20/36) and time constraints (30.6%, 11/36) were cited as barriers. Higher self-rated knowledge correlated positively with efforts to enhance placebo effects or to mitigate nocebo effects through communication (placebo: rho = 0.321; nocebo: rho = 0.451; both p < 0.001).


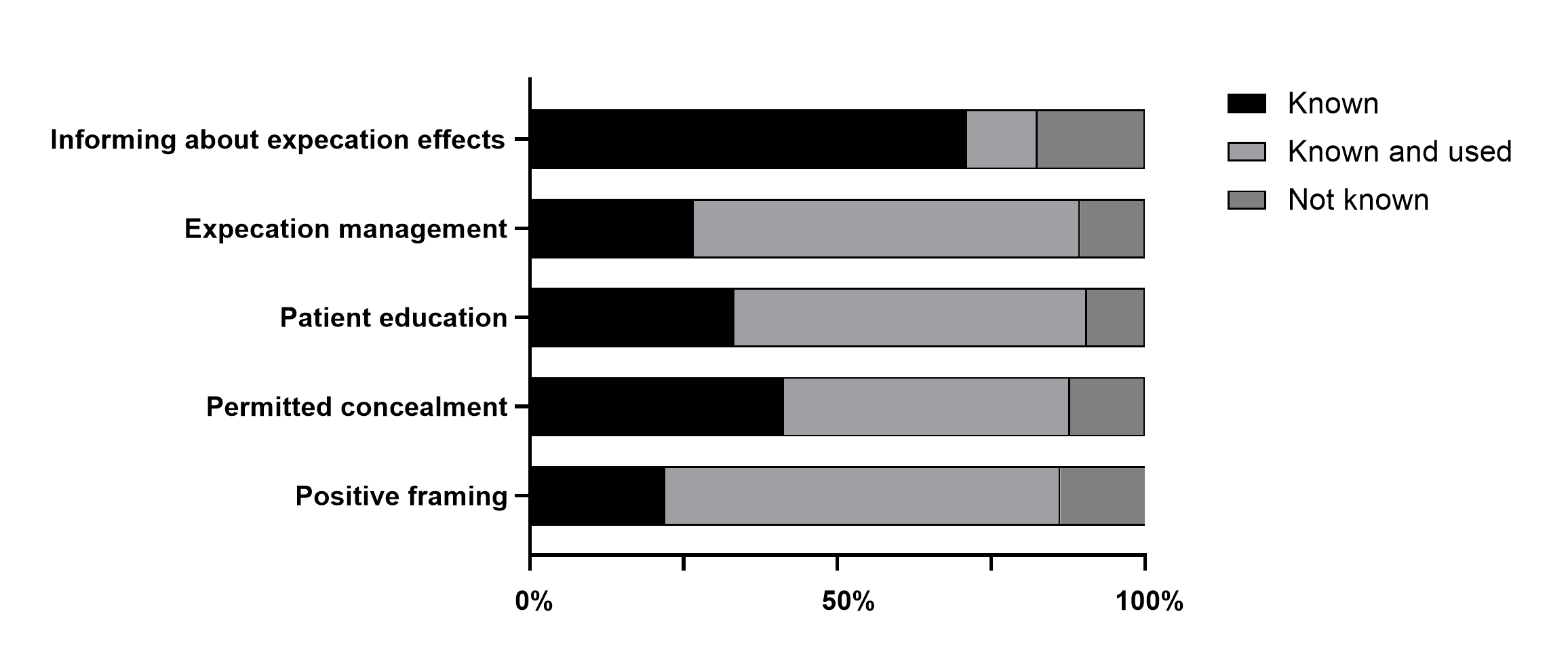


Figure 4: **Knowledge and clinical usage of communication strategies to enhance placebo effects and to avoid nocebo effects.** The figures given in the diagrams are percentages, N = 436.

**Questionnaire: Placebo and nocebo - expectation effects in anaesthesiology**

Dear colleagues,

We would like to invite you to take part in a survey conducted by the Collaborative Research Centre "Treatment Expectations" ([Protected link](https://treatment-expectation.de/)). The aim of the survey is to determine anaesthesiologists’ knowledge about and attitudes towards placebo and nocebo effects in clinical practice. We are referring here to specific expectation effects in the psychosocial treatment context in the area of anaesthesiology.

We would be delighted if you would complete the following short questionnaire. Your participation will make an important contribution to supporting our research and to understanding how expectation effects are used in clinics for the benefit of patients. We thank you in advance for your personal commitment and look forward to presenting you with interesting results.

It will take about 5-10 minutes to fill out the questionnaire.

This survey is supported by the scientific working group "Pain Medicine" of the DGAI.

We thank you for your time and support.

On behalf of the entire team,

Dr med Jana Aulenkamp

Essen University Hospital

Clinic for Anaesthesiology and Intensive Care Medicine

**Part A: Declaration of consent**

Dear participant,

Your data will be treated confidentially and will be collected and analysed in anonymised form. The demographic information requested (e.g. age or gender) does not allow any direct conclusions to be drawn about your person.

Your participation in this study is voluntary. You are free to terminate your participation at any time during this study without providing reasons and without any disadvantages.

If you have any questions about this survey, please contact the study management:

Dr Jana Aulenkamp [jana.aulenkamp@uk-essen.de](mailto:jana.aulenkamp@uk-essen.de)

**Declaration of consent:**

**"I hereby confirm that I am at least 18 years old, that I have read and understood the information on the consent form, and that I am taking part in the survey voluntarily."**

- No (I will not participate in the study)
- Yes

**Part B: Placebo**

1. **How would you rate your knowledge about placebo effects?**

0= No knowledge at all to 10= Best possible knowledge

1. **Do you agree with the following statements?**

*Please tick the response option that best applies to you.*

*(This question only appears if the individual states that they know what the placebo effect is, single answer)*

- I've heard of the placebo effect, but I couldn't put it into words very well.
- I could explain to others in theory what the placebo effect is.
- I could explain to others how the placebo effect can be used in practice.
- I could explain to others how the placebo effect can be used in practice and apply my knowledge in a targeted manner in my clinical practice.

(if ticked here, then a)

- 1. **You have just stated that your theoretical knowledge about the placebo effect has an influence on your practical work. Could you briefly outline what measures you take to specifically utilise the placebo effect?**

*Free text input*

*(This question only appears if the individual states that the placebo effect has an influence)*

1. **Where does your knowledge about the placebo effect come from?**

*Please tick all the response options that apply to you.*

*(This question only appears if the individual states that they know what the placebo effect is)*

- Medical school
- Residency/specialist medical training
- In-service training
- Exchange with colleagues
- Specialist literature (journals/books/online)
- Media/television/internet
- Self-study
- Other
- I don't remember

**Placebo Quiz**

1. **Please indicate to what extent you consider the following statements on the placebo effect to be true**

*0= Does not apply at all|10 = Applies completely*

- Placebo effects occur in all patients.
- Placebos only have an effect if patients firmly believe in them.
- The placebo effect shows clear gender differences.
- Placebos only work as long as patients do not know that they are receiving a placebo.
- The placebo effect is triggered, among other things, by previous experience.
- Placebo effects have a stronger impact on subjective symptoms than on objective symptoms.
- Empathy and affection reinforce the placebo effect.

**Part C: Nocebo**

1. **How would you rate your knowledge about nocebo effects?**

0 = No knowledge at all to 10 = Best possible knowledge

1. **Do you agree with the following statements?**

*Please tick the response option that best applies to you.*

*(This question only appears if the individual states that they know what the nocebo effect is, single answer)*

- I've heard of the nocebo effect, but I couldn't put it into words very well.
- I could explain to others in theory what the nocebo effect is.
- I could explain to others how the nocebo effect can be taken into consideration in practice.
- I could explain to others how the nocebo effect can be taken into consideration in practice and apply my knowledge about the nocebo effect in a targeted manner in my clinical practice.

(if ticked here, then a)

- 1. **You have just stated that your theoretical knowledge about the nocebo effect has an influence on your practical work. Could you briefly outline what measures you take to avoid nocebo effects?**

*Free text input*

*(This question only appears if the individual states that the placebo effect has an influence)*

1. **Where does your knowledge about the nocebo effect come from?**

*Please tick all the response options that apply to you.*

*(This question only appears if the individual states that they know what the placebo effect is)*

- Medical school
- Residency/specialist medical training
- In-service training
- Exchange with colleagues
- Specialist literature (journals/books/online)
- Media/television/internet
- Self-study
- Other
- I don't remember

**Nocebo Quiz**

1. **Please indicate to what extent you consider the following statements on the nocebo effect to be true**

*0= Does not apply at all|10 = Applies completely*

- Nocebo and placebo effects never occur simultaneously.
- The nocebo effect can be described as the absence of a placebo effect.
- The type of communication with the patient can foster the occurrence of a nocebo effect.
- Even in placebo arms of clinical trials, study participation can be discontinued due to undesirable side effects.
- Nocebo effects mainly occur in patients with pre-existing mental illnesses.
- Adverse drug effects are influenced by learning effects.

**Part D: Quick overview of placebo/nocebo**

Classically, a placebo is understood as a treatment without a pharmacologically active ingredient (e.g. a sugar pill) that is administered to the control group in a drug trial in order to determine which improvements in medical symptoms can be attributed solely to the novel drug. In this case, symptom improvements are to be expected in both groups, and the improvements that occur in the placebo group - and therefore cannot be attributed to a pharmacological drug effect - are referred to as the placebo effect. In part, the placebo effect can be attributed to factors such as spontaneous remission and natural fluctuations in the course of a disease. However, it is also partly based on factors that can be influenced, such as doctor-patient communication or characteristics of the patients themselves, like their previous experience and expectations. Nowadays, we know that the placebo effect plays a role not only in the case of sugar pills in drug trials, but also in medical interventions of all kinds.

The nocebo effect might be described as the 'evil twin' of the placebo effect. While a placebo triggers symptom *improvements*, a nocebo causes symptom *deteriorations*. However, just like its 'twin', it does so without pharmacological agents and based on the same factors. In the case of the nocebo effect, these factors can be, for example, previous negative experiences of the patient or warnings about side effects given by the doctor.

**Part E: Relevance of placebo effects**

1. **How appropriate do you consider the targeted utilisation of placebo effects to be in general?**

*Please tick the response option that best applies to you.*

- Utilisation is essential
- Utilisation is acceptable
- Utilisation is only acceptable in rare cases
- Utilisation is never acceptable

1. **How often would you say that you consciously notice a significant improvement due to placebo effects in your everyday clinical work?**

*Please tick the response option that best applies to you.*

- Never
- Rarely
- Sometimes
- Frequently
- Always

1. **How relevant are placebo effects in the following areas of anaesthesiology?** *0= Not at all relevant|10 = Completely relevant*

- Anaesthesia
- Intensive care medicine
- Emergency medicine
- Pain medicine
- Palliative medicine

1. **In which area of work do you consider placebo effects to be particularly relevant?** *Please tick up to 5 response options that apply to you.*

- Informed consent for anaesthesia
- Anaesthesia induction
- Applying a regional anaesthetic
- Anaesthetic withdrawal
- Recovery room
- Postoperative ward round
- Inpatient ward round
- Intervention/puncture
- Discussion with relatives
- Emergency treatment as an emergency doctor
- Shock room treatment
- Treatment of acute pain
- Treatment of chronic pain

1. **For which symptoms do you consider the targeted use of placebo effects to be particularly relevant?**

*Please tick up to 5 response options that apply to you.*

- Fear
- Delirium
- Depression
- Sleep disorder
- Gastrointestinal complaints
- Nausea
- Infections
- Acute pain
- Chronic pain
- Fatigue
- Addiction
- Withdrawal symptoms
- Other
- None

**Part F: Communication**

1. **Do you pay attention to your wording when talking to patients in order to reinforce placebo effects or avoid nocebo effects?**

0 = Never to 10 = Always

1. **What specific communication techniques to reinforce placebo effects or avoid nocebo effects** **do you know of?**

*Please select from: I don’t know of it/ I know of it/ I know of and actively use it*

- Positive framing
- Permitted concealment
- Patient education
- Expectation management
- Information about placebo and nocebo effects

1. **How feasible do you think it is** **to use targeted measures to reinforce placebo effects / avoid nocebo effects in your professional environment?**

*Please tick the response option that best applies to you.*

- Very feasible
- Rather feasible
- Rather not feasible
- Not feasible at all

1. **Why do you consider such communication techniques to be unfeasible in your professional environment?**

*Please tick the response option that best applies to you.*

*(This question only appears if the individual states that they consider the communication techniques to be unfeasible)*

- Would take too long.
- I don't feel confident in dealing with this topic.
- Not worth the effort, as the conversation doesn't have much influence anyway.
- Not supported by my superiors.
- Not supported by my colleagues.
- Other

**Part G: Clinical application of placebo treatments**

1. **Have you ever openly prescribed or recommended an inactive substance (placebo drug) to your patients?**

- Yes
- No
- I don't want to answer

1. **Have you ever given your patients a preparation/treatment solely to induce the placebo effect (e.g. underdosing, wrong indication, at the patient's request)?**

- Yes
- No
- I don't want to answer

1. **Which of the following medications have you recommended or used in the past year primarily to induce the placebo effect?**

*Please tick all the response options that apply to you.*

*(This question only appears if the individual answered Yes to question 19).*

- Sedatives
- Table salt
- Vitamins
- Herbal substances
- Antibiotics
- Analgesics
- Homeopathic remedies
- Other
- None

1. **Have you ever given your patients an inactive substance covertly (e.g. saline infusion declared as a painkiller)?**

- Yes
- No
- I don't want to answer

1. **Were the patients informed/educated in advance about the possibility of a general use of placebo in the course of treatment?**

*(This question only appears if the individual answered Yes to question 21).*

- Yes
- No

1. **Have you ever noticed colleagues giving an inactive substance covertly (e.g. saline infusion declared as a painkiller)?**

- Yes
- No
- I don't want to answer

1. **What do you personally think about the use of placebo drugs in clinical practice?**

*Please tick the response option that best applies to you.*

- I find it acceptable, even if patients do not know that they are receiving a placebo.
- I only find it acceptable as long as patients know that they are receiving a placebo.
- I don't think that it is acceptable.

1. **Several studies have now shown that the open use of placebos (i.e. placebos without deception, open-label placebo) can lead to clinically relevant improvements. Would you prescribe placebos more frequently if these results could be reproduced in anaesthesiology and the administration of placebos was advocated in guideline recommendations?**

- Yes
- No
- I don't want to answer.

1. **Are you interested in finding out more about placebo and nocebo effects?**

- Yes -> If yes, link to the website and display of a contact email
- No

**Part H: Demographic data**

- How old are you?
- Which gender do you feel you belong to?
- What is your professional position? (Resident physician/junior doctor, Consultant, Senior doctor/head of department, private practice, other)
- In which speciality of anaesthesiology do you see the focus of your daily clinical work? (anaesthesia / intensive care medicine / emergency medicine / pain medicine / palliative medicine)
